# Pharmacokinetics and Safety of Single‐Dose Apraglutide in Individuals with Normal and Impaired Hepatic Function: A Phase 1, Open‐Label Trial

**DOI:** 10.1002/cpdd.70006

**Published:** 2026-01-16

**Authors:** Gerard Greig, Justin Hay, Patricia Valencia, Mena Boules, Tomasz Masior

**Affiliations:** ^1^ Ironwood Pharmaceuticals Inc. Basel Switzerland; ^2^ Ironwood Pharmaceuticals, Inc. Boston MA USA; ^3^ Certara Radnor PA USA

**Keywords:** apraglutide, GLP‐2 analogs, hepatic function, intestinal failure, short bowel syndrome

## Abstract

Intestinal failure‐associated liver disease occurs in 20% to 30% of patients with short bowel syndrome and intestinal failure (SBS‐IF). Apraglutide is a glucagon‐like peptide‐2 (GLP‐2) analog in clinical development for the treatment of patients with SBS‐IF. This study assessed the potential for changes in exposure of apraglutide in individuals with impaired hepatic function versus healthy volunteers. In this Phase 1, open‐label, nonrandomized, single‐dose trial, apraglutide 3.5 mg was administered to participants with moderate hepatic impairment (Child‐Pugh B) or normal hepatic function. Primary pharmacokinetic endpoints were area under the plasma concentration–time curve (AUC) from time 0 to infinity (AUC_inf_) or AUC from time 0 to last quantifiable concentration (AUC_last_) if AUC_inf_ could not be reliably estimated, AUC_0–168 h_, and maximum observed plasma concentration (C_max_). Secondary endpoints included safety and tolerability. Each group comprised eight participants. No increased apraglutide exposure was observed in individuals with moderate hepatic impairment. A lower C_max_ and AUC_inf_ of apraglutide was observed in individuals with moderate hepatic impairment versus those with normal hepatic function (C_max_ = 58.7 vs 71.3 ng/mL; AUC_inf_ = 4086 vs 5351 h ng/mL, respectively). The respective geometric mean ratios were 0.835 and 0.936 for C_max_ and AUC_inf_, and the upper bounds of their 90% confidence intervals indicate that participants with moderate hepatic impairment were not overexposed to apraglutide versus those with normal hepatic function. Adverse events were mild or moderate in severity. The results of this trial suggest that apraglutide does not require dose alteration in patients with mild and moderate hepatic impairment.

Short bowel syndrome (SBS) is a rare, severe, and debilitating condition primarily resulting from surgical resection of large portions of the small intestine due to gastrointestinal (GI) damage caused by a range of conditions, including inflammatory bowel disease, ischemia, neoplasms, or physical/abdominal trauma.^1^, ^2^, ^3^, ^4^ The severity of the condition is based on the absorptive capacity of the remnant bowel. The spectrum of severity ranges from intestinal insufficiency (SBS‐II), for which no intravenous supplementation is needed to maintain health, to intestinal failure (short bowel syndrome and intestinal failure [SBS‐IF]), in which the gut function is reduced below the minimum necessary for survival.^4^, ^5^ Patients with SBS‐IF are unable to maintain nutrition needs or fluid, electrolyte, or micronutrient balances when on a conventionally accepted, normal diet and as a result are dependent on parenteral support (PS).^6^ Chronic use of PS is associated with serious and sometimes fatal complications, including catheter‐related bloodstream infections, sepsis, catheter‐related cerebral venous/deep vein thrombosis, and progressive cholestatic liver disease or intestinal failure‐associated liver disease (IFALD), leading to high rates of emergency department visits and inpatient hospitalizations.^6^, ^7^, ^8^, ^9^, ^10^ As a therapeutic modality, PS can also cause disturbances to the gut microbiome.^11^, ^12^ These disturbances, coupled with intestinal dysbiosis and bacterial translocation, are linked to the development of IFALD in 20% to 30% of patients with SBS‐IF.^6^, ^11^, ^12^

Glucagon‐like peptide‐2 (GLP‐2) is an essential endocrine peptide naturally secreted by the intestine to maintain GI function, including nutrient/fluid absorption and barrier function.^13^, ^14^ Its major physiological actions include stimulation of enterocyte proliferation and intestinal perfusion, inhibition of enterocyte apoptosis, maintenance of intestinal barrier function, and regulation of GI motility.^13^, ^14^ GLP‐2 also has regenerative capacity with intestinotrophic effects and has been shown to preserve the global homeostatic environment of the intestinal microbiota.^13^, ^14^, ^15^ GLP‐2 analogs have been developed as a therapeutic option for patients with SBS‐IF,^16^ based on the observation that exogenous GLP‐2 enhances fluid and nutrient absorption and reduces malabsorption and diarrhea in both preclinical and clinical SBS settings.^17^ This, in turn, may reduce or eliminate the need for PS in patients with SBS‐IF.

Apraglutide is a synthetic GLP‐2 analog with a unique pharmacokinetic (PK) profile that is under clinical development for the treatment of SBS‐IF. It has been designed to be longer acting than native GLP‐2, resulting in enhanced intestinotrophic effects.^18^ Apraglutide differs from native human GLP‐2 by four amino acid substitutions that confer unique PK properties; this translates into low systemic clearance in rat models, mostly by increasing plasma protein binding.^3^, ^19^ Preclinical studies show that the structure of apraglutide results in comparable in vitro GLP‐2 receptor potency and selectivity compared with human GLP‐2^3^; a terminal half‐life of approximately 72 h, making it suitable for once‐weekly dosing; and greater intestinotrophic effects in animal models versus the GLP‐2 analogs teduglutide or glepaglutide.^19^, ^20^, ^21^

Apraglutide has also been investigated for the treatment of graft‐versus‐host disease (GvHD), which is a common complication of allogeneic hematopoietic stem cell transplants.^3^, ^18^, ^22^ Due to the high prevalence of hepatic dysfunction in patients with SBS‐IF and those with GvHD, it is important to explore the impact of hepatic impairment on the PK of apraglutide.^11^, ^22^

A Phase 1 study investigating apraglutide in individuals with renal impairment demonstrated that they were not at higher risk of overexposure to apraglutide than those with normal renal function, and that apraglutide dose did not need to be reduced in individuals with renal impairment.^21^

This study assessed the potential for apraglutide overexposure in individuals with moderate hepatic impairment by assessing the PK of apraglutide following a single subcutaneous (SC) dose of 3.5 mg in individuals with moderate hepatic impairment compared with matched controls with normal hepatic function.

## Methods

### Statement of Ethics

The protocol, proposed participant information, informed consent form, and all other applicable trial documents were reviewed and approved by an independent ethics committee; the relevant competent authority was notified and approval was obtained where required (NCT05706623).

This trial was performed in compliance with: Declaration of Helsinki (revised version of Fortaleza, Brazil, 2013); the ICH‐GCP guidelines (E6 R2, November 2016); European Union Clinical Trials Regulation (EU) No. 536/2014; any amendments to these regulations; and local laws and regulations.

All participants enrolled in the trial were provided with sufficient information to make an informed decision about their participation, and formal consent was obtained before an individual participated in any trial‐specific procedures.

### Study Design

This Phase 1, two‐part, open‐label, nonrandomized, single‐dose trial evaluated the effect of impaired hepatic function on the PK, safety, and tolerability of a single dose of apraglutide administered by SC injection in the abdomen (Figure S1). The study consisted of two parts: Part 1 assessed the PK of apraglutide in individuals with moderate hepatic impairment compared with matched healthy individuals with normal hepatic function; and Part 2 was designed to assess the PK of apraglutide in individuals with mild hepatic impairment compared with matched control individuals, only if Part 1 showed increased exposure in individuals with moderate hepatic impairment. The study aimed to evaluate the safety and tolerability of apraglutide administered in individuals with moderate hepatic impairment.

A total of 16 individuals were enrolled in Part 1, including 8 with moderate hepatic impairment and 8 with normal hepatic function, to ensure at least 6 evaluable individuals in each group. Individuals with normal hepatic function were recruited so that the age of each individual was within ±10 years and the body mass index (BMI) was within ±15% of the mean of the individuals in the moderate hepatic impairment group; the groups had an equal number of male and female individuals.

Part 2 was only to be conducted after evaluation of Part 1 if the geometric mean ratio (GMR) point estimate of apraglutide area under the plasma concentration‐time curve (AUC) with terminal phase extrapolated to infinity (AUC_inf_) or AUC from time zero to the last quantifiable concentration (AUC_last_) for the moderate hepatic impairment group compared with the control group was at least two. If the decision criterion to proceed to Part 2 was met, approximately eight individuals with mild hepatic impairment (Child‐Pugh A; Cohort 3) were to be enrolled to ensure at least six evaluable individuals in each group.

The administered dose during the study was 3.5 mg, which was lower than protocol specified dose of 5 mg. This difference was due to dilution effects and injection‐related volume loss during injection preparation and were applied consistently across all injections and participants.

### Participants

Male and female individuals aged 18 to 75 years with a BMI of 18 to 35 kg/m^2^ and a body weight of more than 50 kg were eligible to participate in the study and were assigned to one of the three cohorts based on their hepatic function (estimated by the Child‐Pugh classification). Individuals with hepatic impairment had cirrhosis (due to parenchymal liver disease) and mild (Child‐Pugh A) or moderate (Child‐Pugh B) liver disease that had been clinically stable for at least 1 month before screening (Cohort 1). Healthy individuals had normal hepatic function and no clinically relevant abnormalities in vital signs, electrocardiograms (ECGs), or safety laboratory test results (Cohort 2). When recruiting individuals for the normal and impaired hepatic function groups, the aim was for these groups to be as similar as possible with respect to sex, age, and body weight. The full inclusion/exclusion criteria are shown in Table S1.

### Study Endpoints

The primary study endpoints were the plasma PK parameters of apraglutide. The primary parameters were AUC_inf_, or AUC_last_ if AUC_inf_ could not be reliably estimated; AUC from time 0 to 168 h after apraglutide administration (AUC_0–168 h_); and maximum observed plasma concentration (C_max_). The secondary endpoints were time of maximum plasma concentration (t_max_), apparent clearance after extravascular administration (CL/F), apparent volume of distribution after extravascular administration (V_z_/F), terminal elimination rate constant (k_el_, also known as λ_z_), and terminal half‐life (t_1/2_). The safety endpoints, which were also secondary endpoints, were physical examination findings; adverse events (AEs; incidence, nature, and severity); and changes in clinical laboratory results, vital signs, and 12‐lead ECG during and following trial medication administration.

### PK Assessments

Plasma samples were collected for up to 312 h post dose administration to determine the concentration of apraglutide. Apraglutide was quantified in human plasma samples using a fully validated liquid chromatography mass spectrometry‐based method, with lower and upper limits of quantification of 1 and 200 ng/mL, respectively. Pharmacokinetic parameter calculations were done using Phoenix WinNonlin version 8.1 (Certara USA, Inc.).

### Safety Assessments

An AE was defined as any untoward medical occurrence in an individual administered with a pharmaceutical product that did not necessarily have a causal relationship with the treatment. AEs were collected until the end‐of‐trial visit. The severity of AEs was graded using the National Cancer Institute Common Terminology Criteria for Adverse Events (NCI‐CTCAE) scale. Treatment‐emergent adverse events (TEAEs) were defined as AEs that occurred after dosing the participants with the trial drug. An AE of special interest (AESI), serious or nonserious, was an AE of scientific and medical concern specific to the sponsor's product or program for which ongoing monitoring, additional information, and rapid communication by the investigator to the sponsor could be appropriate. The following were considered AESIs in this study: injection site reactions; GI obstruction; gallbladder, biliary, and pancreatic disease; fluid overload; malignancies; and systemic hypersensitivity. A serious adverse event (SAE) was any untoward medical occurrence at any dose that resulted in death, was life‐threatening, required hospitalization, resulted in persistent or significant disability/incapacity, was a congenital anomaly, or was an important medical event.

### Statistical Methods

#### Sample Size

The sample size of approximately eight individuals per group was also based on the feasibility of recruiting individuals with various degrees of hepatic impairment. No formal power calculation was performed, and the number of individuals per group was based on a review of the literature and US Food and Drug Administration and European Medicines Agency guidelines.

#### Population for Analysis

The safety analysis set (SAS) comprised all individuals who received at least one dose of apraglutide, and all safety analyses were based on the SAS. The PK concentration set was a subset of individuals in the SAS who had at least one PK sample collected, and all PK concentration summaries were conducted using the PK concentration set. The PK parameter set was a subset of individuals in the SAS who had at least one PK parameter of interest estimated, and all PK parameter summaries and primary analyses were conducted using the PK parameter set.

### Statistical Analysis

In Part 1, analysis of variance (ANOVA) was used to compare the natural log‐transformed AUC_inf,_ or AUC_last_ if AUC_inf_ could not be calculated, and C_max_ for apraglutide between the normal hepatic function group (reference) and the moderate hepatic impairment group (test). Estimates of the mean differences (test/reference) and corresponding 90% confidence intervals (CIs) were obtained from the model. The mean differences and 90% CIs for the differences were exponentiated to provide estimates of the GMR (test/reference) and 90% CIs for the ratios.

Part 2 was to be conducted if the GMR for AUC_inf_ (or AUC_last_ when AUC_inf_ could not be calculated) in the moderate hepatic impairment group versus the normal group was at least two. As this criterion was not met, the trial stopped after Part 1, as per protocol. ANOVA was planned to be used in Part 2 to compare the natural log‐transformed AUC_inf_ or AUC_last_ and C_max_ for apraglutide between the normal hepatic function group from Part 1 (reference) and the mild hepatic impairment group (test). Estimates of the mean differences (test/reference) and corresponding 90% CIs were to be obtained from the model. The mean differences and 90% CIs for the differences were to be exponentiated to provide estimates of the GMR (test/reference) and 90% CIs for the ratios.

## Results

### Patient Disposition and Baseline Characteristics

The study was conducted between February 7, 2023 and April 4, 2023. A total of 23 individuals were enrolled in the trial, 15 with normal hepatic function and 8 with moderate hepatic impairment. Of the individuals with normal hepatic function, seven did not receive apraglutide due to withdrawal of consent (n = 4), failure to meet eligibility criteria (n = 2), and withdrawal following a medical decision (history of drug hypersensitivity; n = 1). Therefore, a total of 16 individuals were included in Part 1 of the study: 8 individuals with moderate hepatic impairment and 8 matched individuals with normal hepatic function. All participants were White and were not of Hispanic or Latino ethnicity; 10 (62.5%) were male, and 6 (37.5%) were female. Individuals in the normal and moderately impaired hepatic function groups were well matched in terms of sex and mean age, height, weight, and BMI (Table 1).

**Table 1:** Baseline characteristics

	Characteristics	Normal Hepatic Function (N = 8)	Moderate Hepatic Impairment (N = 8)	Overall (N = 16)
Sex, n (%)	Female	3 (37.5)	3 (37.5)	6 (37.5)
	Male	5 (62.5)	5 (62.5)	10 (62.5)
Race, n (%)	White	8 (100)	8 (100)	16 (100)
Ethnicity, n (%)	Not Hispanic or Latino	8 (100)	8 (100)	16 (100)
Age (years)	Mean	58.3	58.8	58.5
	Min–max	41–71	42–75	41–75
Weight (kg)	Mean	80.3	82.3	81.3
	Min–max	62.2–98.7	51.8–98.5	51.8–98.7
Height (cm)	Mean	171.4	171.6	171.5
	Min–max	162–176	156–184	156–184
BMI (kg/m^2^)	Mean	27.4	27.7	27.6
	Min–max	20–33.9	19–30.9	19–33.9

BMI, body mass index; max, maximum; min, minimum; N, number of individuals randomized; n, number of individuals in group; SD, standard deviation.

### PK Evaluation: Plasma PK Parameters of Apraglutide

**Figure 1:**
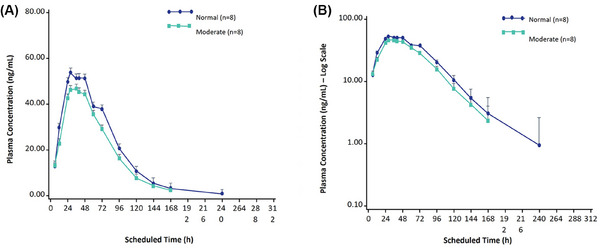
Individual plasma apraglutide concentration versus time in individuals with normal hepatic function (linear scale; pharmacokinetic concentration set).

All 16 individuals were included in the PK analysis. Exposure to apraglutide was lower in individuals with moderately impaired hepatic function (geometric mean AUC_inf_ = 3744 h ng/mL; geometric CV = 50.2%) compared with those with normal hepatic function (geometric mean AUC_inf_ = 4481 h ng/mL; geometric CV = 64.5%) (Table 2). A single dose of apraglutide 3.5 mg achieved a geometric mean C_max_ of 54.6 ng/mL (geometric CV = 43.1%) in individuals with moderately impaired hepatic function and 58.3 ng/mL (geometric CV = 68.3) in individuals with normal hepatic function. Median apraglutide t_max_ in both groups was 32 h before concentrations showed an initial rapid decline, followed by slow elimination in both groups, with a mean CL/F of 1.49 L/h in individuals with moderate hepatic impairment and 1.26 L/h in individuals with normal hepatic function. The plasma PK parameters for apraglutide are shown in Table 2 **and** Table S2. Individual values for C_max_, AUC_inf_, and AUC_0–168 h_ from one participant with normal hepatic function were notably higher than those from the other individuals in the same group (see outlier curve on Figure 1). These individual PK parameter values increased the mean C_max_ and AUC values of this group (Figure 1).

**Table 2:** Summary of apraglutide plasma PK parameters (PK parameter set)

	Normal Hepatic Function Group (N = 8)	Moderate Hepatic Impairment Group (N = 8)
C_max_ (ng/mL)		
CV (%)	83.6	41.4
Geometric mean (geometric CV [%])	58.3 (68.3)	54.6 (43.1)
t_max_ (h)		
Median (minimum–maximum)	32.03 (27.97–48.15)	31.76 (12.00–36.00)
AUC_inf_ (h ng/mL)		
CV (%)	75.0	40.5
Geometric mean (geometric CV [%])	4481 (64.5)	3744 (50.2)
AUC_last_ (h ng/mL)		
CV (%)	76.4	41.1
Geometric mean (geometric CV [%])	4392 (65.6)	3662 (51.3)
AUC_0–168_ (h ng/mL)		
CV (%)	77.1	40.8
Geometric mean (geometric CV [%])	4271 (65.2)	3605 (50.2)
t_1/2_ (h)		
CV (%)	53.7	55.9
Geometric mean (geometric CV [%])	33.2 (51.6)	29.2 (44.4)
CL/F (L/h)		
CV (%)	45.6	54.5
Geometric mean (geometric CV [%])	1.12 (64.5)	1.34 (50.2)
V_Z_/F (L)		
CV (%)	58.8	63.2
Geometric mean (geometric CV [%])	53.4 (91.1)	56.2 (69.4)

AUC_0‐168 h_, area under the plasma concentration‐time curve from time zero to 168 h post dose; AUC_inf_, area under the plasma concentration‐time curve with terminal phase extrapolated to infinity; AUC_last_, area under the plasma concentration–time curve from time zero to the last quantifiable concentration; CL/F, apparent clearance after extravascular administration; C_max_, maximum observed plasma concentration; CV, coefficient of variation; t_1/2_, terminal half‐life; t_max,_ time of maximum plasma concentration; Vz/F, apparent volume of distribution after extravascular administration.

**Table 3:** Analysis of variance of PK parameters (PK parameter set)

		Geometric LS Mean			LS Mean Ratio (Test/Reference)
Comparison (Test vs Reference)	PK Parameter	Test	Reference	Estimate	90% CI [Lower, Upper]
Moderate hepatic impairment versus normal hepatic function	AUC_inf_ (h µg/mL) C_max_ (ng/mL)	374454.6	448158.3	0.83540.9356	[0.5216, 1.3380][0.5889, 1.4864]

AUC_inf_, AUC with terminal phase extrapolated to infinity; C_max_, maximum plasma concentration; LS, least squares; PK, pharmacokinetics.

The analysis was performed on natural log–transformed parameters using an analysis of variance model with fixed effect for treatment and baseline and random effect for individual.

### Apraglutide Concentrations in Plasma Over Time

**Figure 2:**
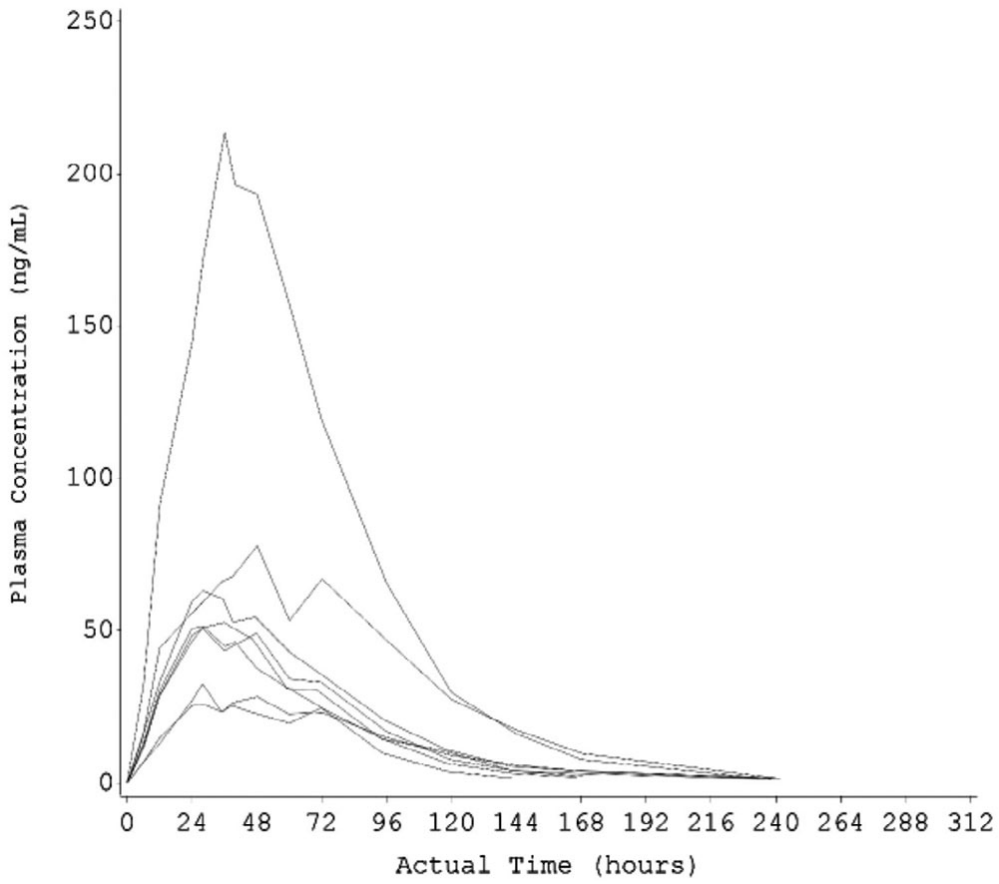
Geometric mean plasma apraglutide concentration–time curves: linear (A) and semi‐log scale (B) (pharmacokinetic concentration set).

Plasma apraglutide concentrations were quantifiable in nearly all individuals at 168 h post dose; by 312 h post dose, concentrations were below the lower limit of quantification in seven of the eight individuals in each group. Plasma apraglutide concentrations in one individual with normal hepatic function were higher than those observed in the other individuals in the group, increasing the mean plasma concentration values of this group across multiple time points (Figure 2). Individuals with moderate hepatic impairment did not have increased apraglutide exposure.

### ANOVA of C_max_ and AUC_inf_

The respective GMRs for AUC_inf_ and C_max_ were 0.835 and 0.936, and the upper bounds of their 90% CIs were less than two (Table 3). Part 2 of the study was only to be conducted if the GMR point estimate for apraglutide AUC_inf_ (or AUC_last_) for Cohort 1 versus Cohort 2 was at least two. As this criterion was not met, the trial was stopped after Part 1.

In a post hoc analysis excluding the individual with normal hepatic function who had extremely high plasma apraglutide concentrations and the paired individual with moderate hepatic insufficiency, the point estimate and 90% CI for the GMR (test/reference) of the AUC_inf_ of apraglutide was 0.959 (0.628, 1.466), and the upper bound of the 90% CI for C_max_ and AUC_inf_ was also below two (Table S3).

### Safety

All of the 16 study individuals (100%) were included in the safety analysis. Three TEAEs were reported for one individual in Cohort 1; no TEAEs were reported for Cohort 2. Two of the TEAEs were of mild severity (headache and pruritus) and one of moderate severity (pruritus); all were considered related to apraglutide. No SAEs or AEs leading to discontinuation were reported in either cohort (Table 4).

**Table 4:** TEAEs (safety analysis set)

	Normal Hepatic Function Group (N = 8)	Moderate Hepatic Impairment Group (N = 8)
Individuals with ≥1 TEAE, n (%) Number of AEs	0 (0)	1 (12.5) 3
Individuals with ≥1 SAE, n (%)	0 (0)	0 (0)
Individuals with ≥1 TEAE leading to discontinuation, n (%)	0 (0)	0 (0)
Individuals with mild TEAEs, n (%) Number of AEs	0 (0)	1 (12.5) 2
Individuals with moderate TEAEs, n (%) Number of AEs	0 (0)	1 (12.5) 1
Individuals with TEAEs by relationship to study drug related, n (%) Number of AEs	0 (0)	1 (12.5) 3
AEs of special interest, n (%) Number of AEs	0 (0)	1 (12.5) 2

AE, adverse event; SAE, serious adverse event; TEAE, treatment‐emergent adverse events.

The individual with three TEAEs had moderate hepatic impairment (Table 5). The individual had a history of alcoholic liver cirrhosis since 2011 and was positive for hepatitis A. The participant experienced a TEAE of pruritus of moderate severity, which began on the day of apraglutide dosing and had a duration of 5 days. The participant was treated with oral hydroxyzine and dimetindene maleate. The moderate TEAE of pruritus resolved with sequelae and immediately after this, a mild TEAE of pruritus was reported. The event of mild pruritus was ongoing at the time of reporting. The two events of pruritus were classed as hypersensitivity AESIs. No other AESIs were experienced by participants during the trial. No TEAEs were reported in individuals with normal hepatic function, including the individual who had a notably higher C_max_ than others in the group.

**Table 5:** Summary of TEAEs by system organ class

System Organ Class Preferred Term^*^	Normal Hepatic Function Group (N = 8) n (%) [E]	Moderate Hepatic Impairment Group (N = 8) n (%) [E]	Total (N = 16) n (%) [E]
Subjects with any TEAE	0	1 (12.5) [3]	1 (6.3) [3]
Nervous system disorders	0	1 (12.5) [1]	1 (6.3) [1]
Headache	0	1 (12.5) [1]	1 (6.3) [1]
Skin and subcutaneous tissue disorders	0	1 (12.5) [2]	1 (6.3) [2]
Pruritus	0	1 (12.5) [2]	1 (6.3) [2]

E, number of events; N, number of individuals exposed; n, number of individuals who experienced the adverse event; TEAE, treatment‐emergent adverse event.

* System Organ Class and Preferred Term per Medical Dictionary for Regulatory Activities (MedDRA).

There was no indication that a single SC dose of apraglutide in individuals with normal or moderately impaired hepatic function led to any clinically meaningful findings in laboratory assessments, vital signs, 12‐lead ECG, or physical findings.

## Discussion

Previous pharmacodynamic and PK studies have demonstrated that apraglutide has unique pharmacokinetic properties resulting in a longer half‐life than SC‐injected native GLP‐2 and other GLP‐2 analogs.^3^, ^20^ The objectives of the present study were to evaluate the PK and tolerability of a single dose of apraglutide 3.5 mg in individuals with normal versus moderately impaired hepatic function. Apraglutide is under clinical development for the treatment of SBS‐IF, which may lead to IFALD, and GvHD, which typically manifests as injury to the skin, GI mucosa, and liver.^3^, ^19^ It was therefore important to assess whether there was overexposure to apraglutide in individuals with mild or moderately impaired hepatic function following a single 3.5‐mg dose, to ensure that similar doses of apraglutide can be administered to patients regardless of hepatic function.

The design of this single‐dose study was adequate as apraglutide exhibits dose‐proportional and time‐independent PK at the concentrations studied. In drugs with such PK characteristics, the AUC_inf_ after a single dose is a good approximation of the AUC over a dosing interval at steady state. The choice of an open‐label design is also relevant as the endpoints were PK endpoints. The actual administered dose during the study of 3.5 mg was lower than protocol specified dose of 5 mg for participants weighing more than 50 kg. This systematic difference was due to dilution effects and injection‐related volume loss during injection preparation. The once‐weekly 3.5‐mg dose is the dose recommended in therapeutic studies for individuals weighing more than 50 kg and was based on prior clinical trials investigating apraglutide in patients with SBS‐IF.^19^, ^21^, ^22^, ^23^, ^24^, ^25^, ^26^ The metric for deciding to proceed to Part 2 of the study was an increase by 100% in the AUC_inf_ and is supported by the literature.

Exposure to apraglutide was lower in individuals with moderately impaired hepatic function than in those with normal hepatic function (geometric mean AUC_inf_ = 3744 vs 4481 h ng/mL, respectively). However, the upper bound of the 90% CI for the geometric least squares mean ratio (test/reference) of AUC_inf_ was less than two, indicating that individuals with moderate hepatic impairment did not risk unreasonable overexposure to apraglutide due to this comorbidity. Therefore, it was not necessary to conduct Part 2 of the study in individuals with mild hepatic impairment, and the trial was stopped at the end of Part 1. The C_max_ of apraglutide was also shown to be lower in individuals with moderately impaired hepatic function compared with those with normal hepatic function (geometric mean C_max_ = 54.6 vs 58.3 ng/mL, respectively). A single 20‐mg SC dose of teduglutide was reported to have a lower C_max_ and AUC (10%–15%) in individuals with moderate hepatic impairment compared with healthy matched control individuals.^27^

Individuals with lower body weight have a lower volume of distribution and a slower clearance of apraglutide, leading to AUC increases with decreasing body weight independent of dose.^21^ In this trial, the healthy participant with the highest plasma apraglutide concentrations had the lowest body weight of 62.2 kg among the healthy participants, and their clearance and volume of distribution were the lowest in this study (data not shown).

Apraglutide was well tolerated in individuals with moderate hepatic impairment and normal hepatic function. All TEAEs were mild or moderate in severity, and no deaths or SAEs were reported during the study. One individual with moderate hepatic impairment experienced two events of pruritus, considered an AESI. However, this individual had a history of alcoholic liver cirrhosis, and pruritus is a well‐recognized symptom of liver cirrhosis due to the deposition of bile salts in the skin.^28^ Overall, the safety was consistent with previous studies evaluating the safety of apraglutide.^19^, ^20^, ^21^

Therapeutic innovations that address significant malabsorption/fluid loss may provide clinical benefit in patients with SBS‐IF, for which the care approach currently includes PS and symptomatic management. Although PS maintains hydration and nutrition status of the patient, it is invasive and time‐consuming, which negatively affects health‐related quality of life, and is associated with serious complications such as bacterial infections, blood clots, kidney disease, and liver problems.^19^ Apraglutide treatment may help patients improve intestinal absorptive function as demonstrated by the reduction of PS requirements and the achievement of enteral autonomy in some patients in clinical trials. The Phase 3, international, randomized, placebo‐controlled, double‐blind, multicenter STARS trial showed that once‐weekly apraglutide significantly decreased weekly PS volume requirements versus placebo (−25.5% vs −12.5%; *P* = .001) and increased the number of days off PS at Week 24 in the overall population versus placebo (43% vs 27.5% of patients achieved at least one additional day off PS per week, respectively; *P* = .040).^23^ An open‐label extension, STARS Extend, is ongoing (NCT05018286) to evaluate the long‐term safety and tolerability as well as clinical outcomes with apraglutide treatment.^29^

### Limitations

While the results of this study suggest that it is not necessary to reduce the dose of apraglutide in patients with mild or moderate hepatic impairment, certain limitations should be acknowledged. Since apraglutide was not studied in patients with severe hepatic impairment, no recommendations for use in this population can be made. Additionally, only participants weighing ≥50 kg were included in the trial and all participants in the treatment arm were dosed with 3.5 mg of apraglutide. Therefore, the results of this study cannot be extrapolated to any other dosage of apraglutide, to patients weighing <50 kg or to patients with severe hepatic impairment.

## Conclusion

The results of this Phase 1 trial suggest that individuals with moderate or mild hepatic impairment are not at higher risk of overexposure to apraglutide than those with normal liver function and that it is not necessary to reduce the dose of apraglutide in patients with moderate or mild hepatic impairment.

## Author Contributions

The concept and design of the study was conducted by Gerard Greig. Data collection, analysis, and interpretation were performed by Gerard Greig. Gerard Greig, Mena Boules, Justin Hay, Patricia Valencia, and Tomasz Masior contributed equally to the writing and revision of the article. All authors reviewed and approved the final draft.

## Conflicts of Interest

GG is a former consultant for Ironwood Pharmaceuticals. MB, PV, and TM are current employees of Ironwood Pharmaceuticals. JH is a consultant for Ironwood Pharmaceuticals and current employee of Certara.

## Funding

This study was funded by VectivBio AG, Basel, Switzerland.

## Supporting information



Supporting information

## Data Availability

The datasets used and/or analyzed during the current study are available from the corresponding author on reasonable request.
