# Supplementary material for: Pharmacokinetics and Safety of Single‐Dose Apraglutide in Individuals with Normal and Impaired Hepatic Function: A Phase 1, Open‐Label Trial
Source: Clin Pharmacol Drug Dev. 2026 Jan 16;15(1):e70006. doi: 10.1002/cpdd.70006 (PMC12811208; doi:10.1002/cpdd.70006)
Supplement: Supplementary file 1 — Supporting information [file CPDD-15-0-s001.docx]

**Pharmacokinetics and safety of single-dose apraglutide in individuals with normal and impaired hepatic function: a phase 1, open-label trial**

Gerard Greig, MD, PhD,*^1^ Justin Hay, PhD,^2,3^ Patricia Valencia, PharmD,^2^ Mena Boules, MD,^2^ Tomasz Masior, MD,^1^

1. Ironwood Pharmaceuticals Inc, Basel, Switzerland

2. Ironwood Pharmaceuticals, Inc, Boston, MA, USA

3. Certara, Radnor, PA, USA

*Authors were affiliated with Ironwood Pharmaceuticals Inc, Basel, Switzerland, at the time the trial was conducted.

**Corresponding author:** Mena Boules, MD

**Email**: mboules@ironwoodpharma.com

**Address:** Ironwood Pharmaceuticals Inc, 100 Summer Street, Suite 2300 Boston, MA 02110

**Supplementary materials**

**Figure S1. Schematic of study design**


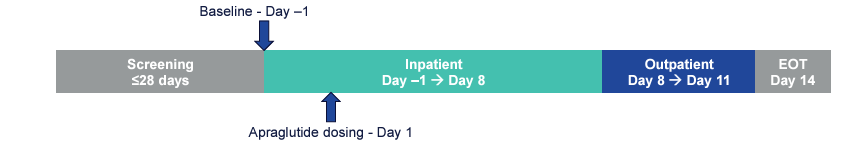


EOT, end of treatment.

**Table S1. Inclusion/exclusion criteria**

| **Inclusion criteria** | **Exclusion criteria** |
| --- | --- |
| ***All participants*** | |
| - Signed and dated ICF prior to any trial-mandated procedure - Male or female aged 18-75 years - BMI of 18-35 kg/m^2^ and a total body weight of >50 kg - Women of childbearing potential on highly effective method of contraception during the trial and for 1 month after the EOT visit - Postmenopausal women. Postmenopausal status was defined as no regular menstrual bleeding for ≥12 months prior to inclusion. Menopause was confirmed by a serum estradiol concentration of <20 pg/mL and a serum FSH level of >40 IU/L - Male individuals with a female partner of childbearing potential committed to practice highly effective methods of contraception and abstain from sperm donation during the trial and for 2 weeks after EOT visit - Able to participate, willing to give written informed consent, and comply with the trial restrictions - Negative result of SARS-CoV-2 polymerase chain reaction testing in the morning prior to admission to the CRU (Day −1) | - History of clinically significant GI, bronchopulmonary, neurological, cardiovascular, endocrine, or allergic disease - Known hypersensitivity to the IMP, any of its excipients, or drugs of the same class - If capable of reproduction, unwilling to use an effective form of contraception - If a female of childbearing potential, a positive urine/blood pregnancy test result - Women who were breast-feeding - Positive urine/blood test for alcohol and drugs of abuse at Screening and on Day  −1 - Use of prohibited medications or herbal remedies - Known presence or history of intestinal polyps or any type of cancer - Pancreatic events such as acute pancreatitis, pancreatic duct stenosis, pancreas infection, and increased blood amylase and lipase (>2.0–5.0 × upper limit of normal range) - Participation in an investigational drug or device trial within 30 days prior to Screening - Donation of blood >500 mL within 2 months prior to screening - Heavy use of tobacco products (ie, smoked >10 cigarettes per day) - Concomitant disease or condition that could have interfered with, or for which the treatment might have interfered with, the conduct of the trial, or that would, in the opinion of the Investigator, have posed an unacceptable risk to the individual in this trial - Any intercurrent clinically significant illness in the previous 28 days before Day 1 of this trial as determined by Investigator - Positive results for HIV antigen/antibody combination, HAV, HBsAgB, or HCV tests. Individuals recovered from hepatitis B or C could be enrolled, ie, they had markers of the infection, but the viral load was undetectable. Individuals with evidence of an acute or chronically active hepatitis B or C infection were excluded. Individuals who were anti-HBsAgB positive, with an undetectable viral load and with history of vaccination against hepatitis B were eligible for enrolment - Unwillingness or inability to comply with the trial protocol for any other reason |
| ***Individuals with normal hepatic function (Cohort 2)*** | |
| - No clinically relevant abnormalities identified by detailed medical history, full physical examination, measurement of heart rate, 12-lead ECG, and clinical laboratory tests | - Any clinically relevant abnormal laboratory test results that were not in line with individual with normal hepatic function status at the discretion of the Investigator - Supine pulse rate <40 or >100 bpm at screening. Supine systolic blood pressure <90 or >140 mm Hg. Supine diastolic blood pressure <45 or >90 mm Hg at screening |
| ***Individuals with impaired hepatic function (Cohorts 1 and 3)*** | |
| - Diagnosis of cirrhosis due to parenchymal liver disease, confirmed and documented by medical history and ≥1 of the following: physical examination, hepatic ultrasonography, computed axial tomography scan, magnetic resonance imaging, and/or liver biopsy - **Cohort 1:** Moderate liver disease (Child-Pugh B) that had been clinically stable (defined as no clinically significant change in disease status) for ≥1 month prior to Screening at the discretion of the Investigator - **Cohort 3:** Mild liver disease (Child-Pugh A) that had been clinically stable (defined as no clinically significant change in disease status) for ≥1 month prior to Screening at the discretion of the Investigator | - Any clinically significant abnormalities not in line with having stable liver disease status - Supine pulse rate <40 or >100 bpm at screening. Supine systolic blood pressure <100 or >170 mm Hg at screening. Supine diastolic blood pressure <60 or >100 mm Hg at screening - Diagnosis of cholestasis and/or gallbladder sludge/stones - History of esophageal bleeding within the last 3 months prior to screening - Severe hepatic encephalopathy (Grade >2) or degree of central nervous system impairment which the Investigator considered sufficiently serious to interfere with the informed consent, conduct, completion, or results of this trial, or constituted an unacceptable risk to the individual - History of liver transplant - Advanced ascites and ascites that required emptying and/or albumin supplementation within 30 days prior to Day 1, or during the course of the trial, as judged by the Investigator - Hemoglobin concentration <100 g/L (10 g/dL) |

BMI, body mass index; bpm, beats per minute; CRU, clinical research unit; ECG, electrocardiogram; EOT, end of trial; FSH, follicle-stimulating hormone; GI, gastrointestinal; HAV, hepatitis A virus; HBsAg, hepatitis B surface antigen; HCV, hepatitis C virus; HIV, human immunodeficiency virus; ICF, informed consent form; IMP, investigational medical product; SARS-CoV-2, severe acute respiratory syndrome coronavirus 2; ULN, upper limit of normal range.

**Table S2. Full summary of apraglutide plasma PK parameters (PK parameter set)**

|  | **Normal hepatic function group  (N=8)** | **Moderate hepatic impairment group  (N=8)** |
| --- | --- | --- |
| **C_max_ (ng/mL)** |  |  |
| Mean (standard deviation) | 71.3 (59.6) | 58.7 (24.3) |
| CV (%) | 83.6 | 41.4 |
| Median (minimum–maximum) | 52.1 (28.3–214) | 59.2 (28.7–108) |
| Geometric mean (geometric CV [%]) | 58.3 (68.3) | 54.6 (43.1) |
| **t_max_ (h)** |  |  |
| Median (minimum–maximum) | 32.03 (27.97–48.15) | 31.76 (12.00–36.00) |
| **AUC_inf_ (h∙ng/mL)** |  |  |
| Mean (standard deviation) | 5351 (4015) | 4086 (1654) |
| CV (%) | 75.0 | 40.5 |
| Median (minimum–maximum) | 3486 (2378–14,503) | 4356 (1571–6648) |
| Geometric mean (geometric CV [%]) | 4481 (64.5) | 3744 (50.2) |
| **AUC_last_ (h∙ng/mL)** |  |  |
| Mean (standard deviation) | 5274 (4028) | 4009 (1647) |
| CV (%) | 76.4 | 41.1 |
| Median (minimum–maximum) | 3751 (2309–14, 461) | 4287 (1512–6559) |
| Geometric mean (geometric CV [%]) | 4392 (65.6) | 3662 (51.3) |
| **AUC_0–168_ (h∙ng/mL)** |  |  |
| Mean (standard deviation) | 5128 (3952) | 3936 (1607) |
| CV (%) | 77.1 | 40.8 |
| Median (minimum–maximum) | 3677 (2312–14,217) | 4163 (1512–6561) |
| Geometric mean (geometric CV [%]) | 4271 (65.2) | 3605 (50.2) |
| **t_1/2_ (h)** |  |  |
| Mean (standard deviation) | 37.0 (19.9) | 32.1 (17.9) |
| CV (%) | 53.7 | 55.9 |
| Median (minimum–maximum) | 27.1 (18.4–75.3) | 27.8 (19.8–74.8) |
| Geometric mean (geometric CV [%]) | 33.2 (51.6) | 29.2 (44.4) |
| **CL/F (L/h)** |  |  |
| Mean (standard deviation) | 1.26 (0.577) | 1.49 (0.810) |
| CV (%) | 45.6 | 54.5 |
| Median (minimum–maximum) | 1.30 (0.345–2.10) | 1.15 (0.752–3.18) |
| Geometric mean (geometric CV [%]) | 1.12 (64.5) | 1.34 (50.2) |
| **V_Z_/F (L)** |  |  |
| Mean (standard deviation) | 66.2 (38.9) | 66.9 (42.3) |
| CV (%) | 58.8 | 63.2 |
| Median (minimum–maximum) | 61.1 (12.3–111) | 44.5 (24.2–139) |
| Geometric mean (geometric CV [%]) | 53.4 (91.1) | 56.2 (69.4) |

AUC_0-168h_, area under the plasma concentration-time curve from time zero to 168 hours post dose; AUC_inf_, area under the plasma concentration-time curve with terminal phase extrapolated to infinity; AUC_last_, area under the plasma concentration-time curve from time zero to the last quantifiable concentration; CL/F, apparent clearance after extravascular administration; C_max_, maximum observed plasma concentration; CV, coefficient of variation; t_1/2_, terminal half-life; t_max,_ time of maximum plasma concentration; Vz/F, apparent volume of distribution after extravascular administration.

**Table S3.** **Post hoc** **analysis of variance of PK parameters excluding the individual with high exposure (PK parameter set)**

|  | | **Geometric LS mean** | | | **LS mean ratio (test/reference)** |
| --- | --- | --- | --- | --- | --- |
| **Comparison**  **(test vs reference)** | **PK parameter** | **Test** | **Reference** | **Estimate** | **90% CI**  **[lower, upper]** |
| **Moderate hepatic impairment**  **vs**  **normal hepatic function** | AUC_inf_ (h·µg/mL)  C_max_ (ng/mL) | 3634  52.8 | 3789  48.5 | 0.9591  1.0888 | [0.6275, 1.4659]  [0.7469, 1.5873] |

AUC_inf_, AUC with terminal phase extrapolated to infinity; C_max_, maximum plasma concentration; LS, least squares; PK, pharmacokinetics.
